# Professor Sir Peter J. Lachmann, FRS, FMedSci (1931–2020)

**DOI:** 10.3390/v13061012

**Published:** 2021-05-28

**Authors:** Richard A. Harrison, B. Paul Morgan

**Affiliations:** 1 School of Medicine, Cardiff University, Cardiff CF14 4XN, UK; 2 Division of Infection and Immunity, School of Medicine, Systems Immunity Research Institute, Cardiff University, Heath Park, Cardiff CF14 4XN, UK; morganbp@cardiff.ac.uk

As members of the International Complement Society (ICS) will be aware, Professor Sir Peter Lachmann sadly passed away, peacefully and at home, on 26th December 2020. Peter, as he was known to all within the Complement Community, was a man of many talents, with a keen interest, deep knowledge and expertise spanning much of medical science. His many appointments and honours have been listed in other eulogies (Christs College; Complement UK). Other than to mention that he was one of the first recipients, in 1997, of the European Complement Network Medal, awarded (with hindsight, rather prematurely) in recognition of his lifetime achievement in the field of Complement, we will not reproduce them here. Instead, we will focus on his immense influence on the Complement field, in a career that spanned over 60 years.

Quite simply, with his passing, the Complement field has lost a giant, someone who played a key role in establishing the foundations on which so many of us have built. Peter entered the Complement field at the beginning of the 1960s, a time when the first efforts to translate the phenomenology that had dominated complement research since its discovery at the end of the 19th century into molecular biochemistry were starting to bear fruit. From the outset, the breadth of his interests was apparent. His early publications included studies of surface deposition of complement components and its consequences in autoimmune disease (1962, with Hans Müller-Eberhard and Henry Kunkel), analysis of conglutinin and immunoconglutinins, leading to the identification and characterisation of conglutinogen activating factor (KAF), now known as Factor I, and with a detailed analysis and characterisation of the components and mechanism of lytic pathway, notably the demonstration of reactive or bystander lysis, critical for understanding membrane attack.

That said, his early work undoubtedly centred on the dissection of phenomena that were associated with dysregulated C3 activation in disease, including nephritic factors and factor I deficiency. Typically, he brought mechanisms to these phenomena, leading, in 1973, to publication of the “C3 tickover hypothesis” [1,2], an elegant hypothesis that established the importance of the C3b feedback cycle as an amplifier of complement activation, validated by multiple lines of investigation over the years, and as relevant in 2021 as it was in 1973. “C3 tickover” provides a perfect example of something that he frequently reminded us—that just because something was discovered several decades ago, and without the benefit of “modern” technology, it is not necessarily irrelevant or wrong. As Peter would often say, we ignore the past (historic literature) at our peril.

Subsequent to this early work on the amplification loop of C3b generation, his groups made substantial contributions to the characterisation of C3 activation and inactivation mechanisms, their regulation, and the properties and pathological relevance of C3 activation products. In the years before his “official” retirement, Peter was also involved in the discovery and characterisation of CD59, and developed a keen interest in roles of complement within the nervous system, something that continues to pose questions and challenges for us today. Post “retirement”, in 1997, Peter’s continued interest in research was manifested within a small group he established at the Department of Veterinary Medicine at the Cambridge Veterinary School, conveniently located just across the road from his home in West Cambridge. This was a very productive period—around 50 of his 338 PubMed-listed publications date from this last research “home”. In truth, Peter never retired, as his boundless energy remained; indeed, at the time of his death he was actively planning a collection of articles focused on the amplification loop or an alternative pathway of complement. An inspiration to us all.

Peter’s research achievements were legion, but to focus just on these would be an injustice to a man who was an inspiration and mentor to many in the field, including the signatories. Within his own research groups at the Medical Research Council Centre in Cambridge he catalysed a vibrant atmosphere, and gave his staff and students licence to think independently and follow their own ideas. Although his core research groups were small in comparison with many, he attracted a constant stream of visitors at all levels of seniority. Space was always found to fit in yet one more person, and the resulting exchange of ideas and stimulating discussions at the bench, unconstrained by seniority, cultivated innovation. Those of us privileged enough to have been a part of this environment will never forget the experience; many others have benefited from Peter’s capacity for mentoring and support; he was always ready with guidance and help, despite his busy schedule.

Peter was a highly influential and involved participant at International Complement Workshops and Meetings from the very start, and a constant presence over the decades. He and his team organised a landmark ICS Conference in Cambridge in 1991. In later years, he was always accompanied by Sylvia, his wonderful wife, a pillar of support throughout his professional life—and, on one infamous occasion, a physical defender when things got “sticky” on the streets of Bari, Italy, during an ECN meeting. While in recent years, attendance at non-UK Meetings became limited by his increasing physical frailty, it was clear that his mental acuity remained as sharp as ever. Many in the field will have memories of being interrogated by Peter after a presentation, frequently the first questioner and usually beginning with “As you well know...”. In more recent years, possibly in recognition of the youth of presenters, this was often tempered to “As you should know...”, or even “As you might know...” or “Let me remind you....”. However phrased, the question or comment that followed was always insightful and pertinent. Often, the bruised presenter would be cornered at the next break by a twinkling Peter, offering enthusiastic congratulations. A wonderful example to us all of how we should actively participate in meetings.

It would be wrong to conclude this tribute without visiting Peter’s personal side. Despite his seemingly total immersion in academic research and leadership, Peter remained very much a family man. It was evident from the way he spoke that he took great pride in the achievements of his wife and children. This interest extended to the families of those he had worked with. Not only did he have an encyclopaedic memory of science, he sometimes seemed to remember more about your own family than you did yourself. Together with Sylvia, he hosted wonderfully relaxed summer parties in the garden of their home in Cambridge, where past and present co-workers could relax, catch up and reminisce. While the fun side of his character was more deeply hidden, it was very definitely there. He would, for example, take time outside of Complement Workshops to visit, often with members of his “Group” in tow, the local sights-from the wonders of the Everglades to the jungles of Brazil.

Peter will be greatly missed, and he will hold a special place in many of our memories. Perhaps the greatest tribute we in the complement field can pay to him will be to ensure that his legacy is not forgotten. That we remain diligent in our pursuit of knowledge, collaborate freely, continue to challenge at meetings, and most important of all, enjoy the privilege of careers that allow us to contribute to current knowledge and understanding. Thank you, Peter, for all that you have taught us.

Richard Harrison and Paul Morgan.
